# Role of electrocardiogram findings in predicting 48-h mortality in patients with traumatic brain injury

**DOI:** 10.1186/s12883-022-02717-y

**Published:** 2022-05-24

**Authors:** Ji Ho Lee, Dong Hun Lee, Byung Kook Lee, Yong Soo Cho, Dong Ki Kim, Yong Hun Jung

**Affiliations:** 1 Department of Emergency Medicine, Chonnam National University Hospital, 42 Jebong-ro, Donggu, Gwangju, Republic of Korea; 2 Department of Emergency Medicine, Chonnam National University Medical School, 160 Baekseo-ro, Dong-gu, Gwangju, Republic of Korea

**Keywords:** Traumatic brain injury, Electrocardiogram, Prognosis

## Abstract

**Objective:**

Electrocardiogram (ECG) patterns can change, especially in patients with central nervous system disorders such as spontaneous subarachnoid hemorrhage. However, the association between the prognosis of traumatic brain injury (TBI) and ECG findings is unknown. Therefore, this study aimed to compare and to analyze ECG findings to predict early mortality in patients with TBI.

**Methods:**

This retrospective observational study included patients with severe trauma and TBI who were admitted to the emergency department (ED) between January 2018 and December 2020. TBI was defined as an abbreviated injury scale score of the head of ≥3. We examined ECG findings, including PR prolongation (≥ 200 ms), QRS complex widening (≥ 120 ms), corrected QT interval prolongation (QTP, ≥ 480 ms), ST-segment elevation, and ST-segment depression (STD) at ED arrival. The primary outcome was 48-h mortality.

**Results:**

Of the total patients with TBI, 1024 patients were included in this study and 48-h mortality occurred in 89 patients (8.7%). In multivariate analysis, QTP (odds ratio [OR], 2.017; confidence interval [CI], 1.203–3.382) and STD (OR, 8.428; 95% CI, 5.019–14.152) were independently associated with 48-h mortality in patients with TBI. The areas under the curve (AUCs) of the revised trauma score (RTS), injury severity score (ISS), QTP, STD, and the combination of QTP and STD were 0.790 (95% CI, 0.764–0.815), 0.632 (95% CI, 0.602–0.662), 0.605 (95% CI, 0.574–0.635), 0.723 (95% CI, 0.695–0.750), and 0.786 (95% CI, 0.759–0.811), respectively. The AUC of the combination of QTP and STD significantly differed from that of ISS, QTP, and STD, but not RTS.

**Conclusion:**

Based on the ECG findings, QTP and STD were associated with 48-h mortality in patients with TBI.

## Introduction

Traumatic brain injury (TBI) contributes to a substantial number of deaths and cases of permanent disability [1]. An estimated 2.8 million people experience TBI annually, leading to death in 50,000 and hospitalization in 282,000. TBI is a contributing factor to 30% of all injury-related deaths in the United States [1]. Owing to advances in trauma care, the risk of death from multiple organ dysfunction syndrome gradually decreases after 48 h [2]. In contrast, deaths within 48 h account for 61.9% of all trauma-related deaths, the causes of which include exsanguination and TBI [2, 3]. Therefore, it is important to assess risk factors early and to provide critical care for patients with a high risk of death within 48 h.

Many triaging tools for TBI have been developed, and several studies have investigated the effectiveness of these tools in predicting outcomes [4, 5]. The injury severity score (ISS) and revised trauma score (RTS) are commonly used tools in trauma, including TBI [4, 5].

TBI-related death is associated with the severity of brain injury, malignant cerebral edema, and extracranial pathologies, especially cardiac electrical dysfunction [6]. Cerebrogenic cardiovascular damage may result in sudden cardiac death through central autonomic dysfunction with elevated catecholamine levels, shift in the ratio of potassium ions to sodium ions after renin–angiotensin system activation, and cerebral injury–related inflammatory responses [6–9]. Thus, cerebrogenic cardiovascular damage, which results in abnormal electrocardiogram (ECG) findings, can have an important association with the outcome of patients with TBI. However, few studies have assessed the relationship between ECG findings and outcome in patients with TBI. Therefore, this study aimed to compare and to analyze the role of ECG findings in predicting early mortality in patients with TBI. Furthermore, this study examined the performance of ECG findings in predicting early mortality compared with previously reported prognostic tools such as RTS and ISS.

## Methods

### Study design and population

In this retrospective observational study, we enrolled patients with severe trauma and TBI who were admitted to the emergency department (ED) of Chonnam National University Hospital, Gwangju, South Korea, between January 2018 and December 2020. Severe trauma was defined as an ISS of > 15 [10]. TBI was defined as an abbreviated injury scale (AIS) score of the head of ≥3 [11]. Isolated TBI was defined as a head AIS score of ≥3 and any other AIS score of < 3 [12]. Combined TBI was defined as a head AIS score of ≥3 and at least one other AIS score of ≥3 [12]. The exclusion criteria were as follows: age < 18 years; burns, hanging, and drowning as specific trauma mechanisms; cardiac arrest after trauma before ED arrival; ECG data not measured or available at ED arrival﻿; and missing data. The institutional review board at Chonnam National University Hospital approved the study protocol.

### Data collection

The following data were obtained during the study period: age, trauma mechanism, sex, preexisting illness (previous percutaneous coronary intervention, hypertension, diabetes, renal impairment, and cerebrovascular accident), respiratory rate (RR), pulse rate, body temperature (BT), Glasgow Coma Scale (GCS) score, systolic blood pressure (SBP), ECG data on ED arrival, emergency operation, and 48-h mortality. RTS was calculated on the basis of the GCS score, SBP, and RR [13]. ISS was calculated on the basis of the AIS score [14]. To determine the presence of massive bleeding, we investigated whether massive transfusion (MT) was provided, which was defined as transfusion of > 10 units of PRCs within the first 24 h of admission or > 4 units in 1 h [15]. The primary outcome in this study was 48-h mortality in patients with TBI.

For analysis, posttrauma ECG data were obtained from the first interpretable 12-lead ECG within 1 h at ED arrival. ECGs were recorded at a speed of 25 mm/s and amplification of 10 mm/mV. We collected the PR interval (ms), QRS interval (ms), QT interval (ms), and ST-segment change as ECG data. The PR interval was the time from the onset of the P wave to the start of the QRS complex. It represents conduction through the atrioventricular node. PR prolongation was defined as a PR interval of ≥200 ms [16]. In patients with myocardial infarction or cardiomyopathy, the presence of heart scar tissue may slow down the electrical conduction between myocardial cells, resulting in widening of the QRS complexes [17]. QRS complex widening was defined as a QRS interval of ≥120 ms [17]. The QT interval was the time from the start point of the QRS complex, expressed as ventricular depolarization, to the return point (visualized) of the T wave, which results from ventricular repolarization. The corrected QT (QTc) interval was calculated after correcting for heart rate with the Bazett formula, as follows: QTc = QT/square root of the RR interval duration [18, 19]. QTc prolongation (QTP) was defined as a QTc interval of ≥480 ms [18, 19]. The ST segment was defined as the interval between ventricular depolarization and repolarization. In this study, we divided the recorded ST-segment changes into ST-segment elevation (STE) and ST-segment depression (STD). STE was defined as an elevation of ≥2 mm in a single lead, and STD was defined as a depression of ≥0.5 mm in two adjacent leads [20].

### Statistical analyses

As continuous variables did not satisfy the normality test, median values are presented as interquartile ranges. Differences between continuous variables were analyzed using the Mann–Whitney *U*-test. Categorical variables are presented as frequencies and percentages. The chi-square test or Fisher’s exact test was used to analyze differences between categorical variables, as appropriate.

The results of multivariate analysis with logistic regression of covariates for 48-h mortality are expressed as odds ratios (ORs) and 95% confidence intervals (CIs). Variables with a *P* <  0.20 in univariate comparisons were included in the multivariate regression model. Using a step-by-step backward approach, variables with a threshold *P* > 0.10 were eliminated from the final adjusted regression model. Receiver operating characteristic curve analysis was performed to evaluate the prognostic performance of RTS, ISS, and ECG findings. The DeLong method was used for comparisons of area under the curve (AUC) values [21]. All analyses were performed using PASW/SPSS™ software (version 18; IBM Inc., Chicago, IL, USA) and MedCalc (version 19.0; MedCalc Software, bvba, Ostend, Belgium). A two-sided *p*-value of 0.05 was used to indicate statistical significance.

## Results

### Patient selection and characteristics

**Fig. 1:**
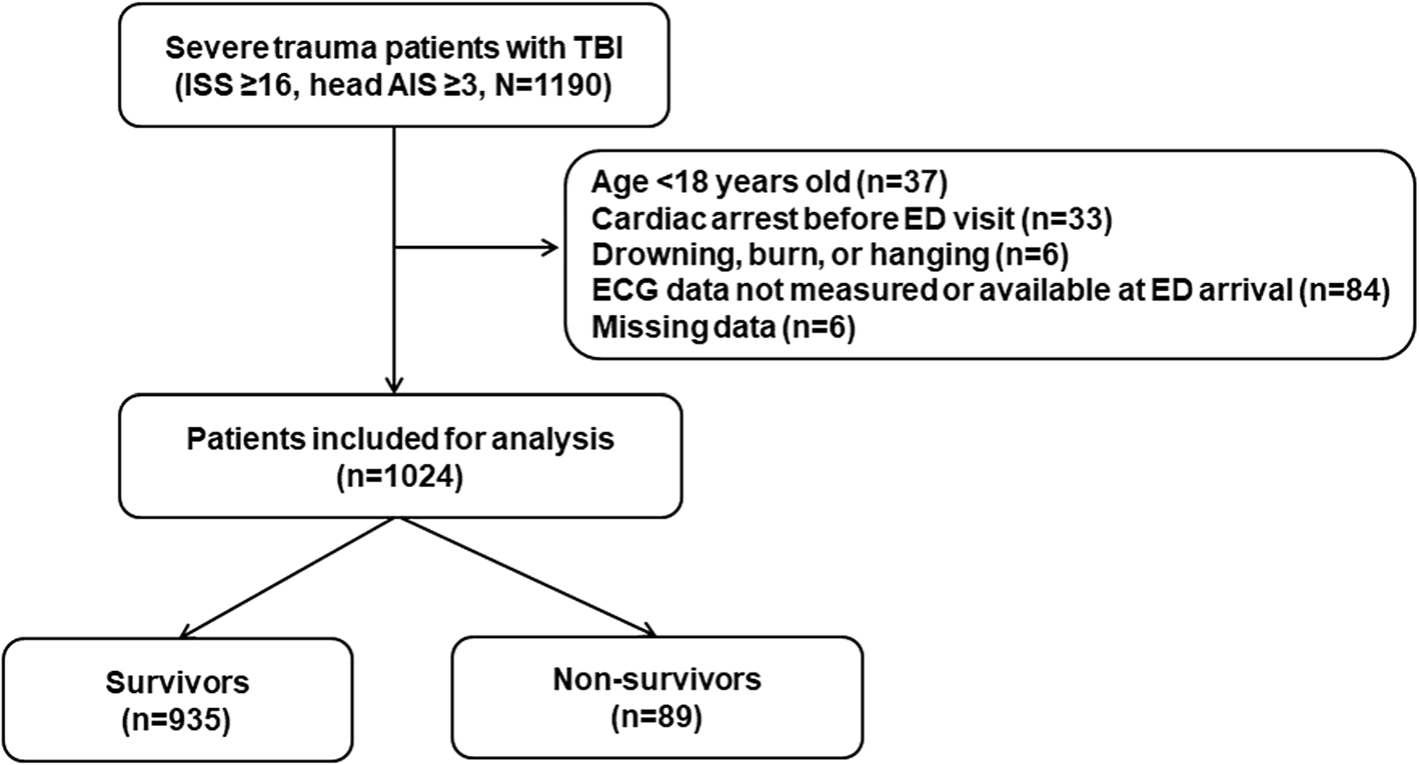
Schematic diagram showing the number of patients with severe trauma included in the present stud. TBI, traumatic brain injury; ISS, injury severity score; AIS, abbreviated injury scale; ED, emergency department; ECG, electrocardiogram

A total of 1190 patients with TBI who met the inclusion criteria were enrolled in this study. After applying the exclusion criteria, 1024 patients were finally included (Fig. 1). The number of male patients was 767 (74.9%), and the median patient age was 63.1 years (52.0–74.0 years). The 48-h mortality rate was 8.7% (*n* = 89). Of the 89 nonsurvivors, 79 (88.8%) died from brain herniation and 10 (11.25) died from massive hemorrhage.

### Comparison of baseline and clinical characteristics between survivors and nonsurvivors

Table 1 compares the characteristics of survivors and nonsurvivors. Nonsurvivors had lower RTS, GCS score, and BT and higher ISS and pulse rate than survivors. No significant difference in SBP was observed between survivors and nonsurvivors. MT was more frequently performed in nonsurvivors than in survivors (Table 1).

**Table 1:** Comparison of baseline characteristics of patients with TBI according to in-hospital mortality within 48 h

Variables	Patients with TBI (*N* = 1024)	Survivors (*n* = 935)	Nonsurvivors (*n* = 89)	*P*
Age, years	63.1 (52.0–74.0)	63.1 (52.0–74.0)	67.0 (53.1–76.5)	0.072
Male sex, n (%)	767 (74.9)	700 (74.9)	67 (75.3)	1.000
Preexisting illness, n (%)				
Previous PCI history	48 (4.7)	46 (4.9)	2 (2.2)	0.380
Hypertension	345 (33.7)	323 (34.5)	22 (24.7)	0.079
Diabetes	221 (21.6)	207 (22.1)	14 (15.7)	0.204
Renal impairment	15 (1.5)	15 (1.6)	0 (0.0)	0.458
Cerebrovascular accident	14 (1.4)	14 (1.5)	0 (0.0)	0.494
Trauma mechanism				1.000
Blunt	1020 (99.6)	931 (99.6)	89 (100.0)	
Penetrating	4 (0.4)	4 (0.4)	0 (0.0)	
Revised trauma score	5.97 (5.03–7.84)	5.97 (5.64–7.84)	4.09 (2.83–5.13)	< 0.001
Injury severity score	22 (16–25)	22 (16–25)	25 (21–26)	< 0.001
Glasgow Coma Scale score	14 (7–15)	14 (8–15)	4 (3–6)	< 0.001
Systolic BP, mmHg	130 (110–140)	130 (110–140)	120 (90–160)	0.259
Respiratory rate, /min	20 (20–20)	20 (20–20)	20 (20–24)	0.002
Pulse rate, /min	84 (74–96)	84 (74–95)	92 (73–108)	0.004
Body temperature, °C	36.4 (36.1–36.8)	36.4 (36.2–36.8)	36.2 (36.0–36.4)	< 0.001
Massive transfusion, n (%)	85 (8.3)	64 (6.8)	21 (23.6)	< 0.001
Emergency operation, n (%)	259 (25.3)	243 (26.0)	16 (18.2)	0.138

*TBI* traumatic brain injury, *PCI* Percutaneous coronary intervention, *BP* blood pressure

A significant difference in PR interval was observed between survivors and nonsurvivors; however, there was no significant difference in the proportion of patients with PR prolongation (Table 2). Moreover, no significant difference was observed in the QRS interval between survivors and nonsurvivors, although the proportion of patients with widened QRS complexes showed a significant difference between groups (Table 1). The QTc interval was more prolonged in nonsurvivors than in survivors (460 [438–480] vs. 479 [456–512] ms, *P* <  0.001). The incidences of STE and STD in nonsurvivors were higher than those in survivors (Table 2).

**Table 2:** Comparison of electrocardiogram variables of patients with TBI according to in-hospital mortality within 48 h

Variables	Patients with TBI (*N* = 1024)	Survivors (*n* = 935)	Nonsurvivors (*n* = 89)	*P*
PR, ms	164 (146–184)	164 (148–186)	152 (135–174)	0.001
PR prolongation, n (%)	143 (14)	134 (14.3)	9 (10.1)	0.349
QRS, ms	92 (84–102)	92 (86–102)	92 (82–104)	0.754
QRS widening, n (%)	77 (7.5)	64 (6.8)	13 (14.6)	0.015
QTc, ms	461 (440–482)	460 (438–480)	479 (456–512)	< 0.001
QTc prolongation, n (%)	288 (28.1)	245 (26.2)	43 (48.3)	< 0.001
Presence of STE, n (%)	84 (8.2)	71 (7.6)	13 (14.6)	0.036
Presence of STD, n (%)	158 (15.4)	108 (11.6)	50 (56.2)	< 0.001

*TBI* traumatic brain injury, *BP* blood pressure, *STE* ST-segment elevation, *STD* ST-segment depression

In the isolated TBI group, survivors had higher RTS, GCS score, and BT values and lower ISS and PR values than non-survivors (Table 3). The PR interval, QTc interval, and incidence rates of QTP and STD were significantly different between survivors and nonsurvivors (Table 4).

**Table 3:** Comparison of baseline characteristics according to in-hospital mortality within 48 h between the isolated TBI and combined TBI groups

Variables	Isolated TBI (*N* = 776)			Combined TBI (*N* = 248)		
	Survivors (*n* = 715)	Non-survivors (*n* = 61)	*P*	Survivors (*n* = 220)	Non-survivors (*n* = 28)	*P*
Age, years	64 (53–74)	68 (55–77)	0.138	60 (50–70)	64 (52–77)	0.212
Male sex, n (%)	536 (75.0)	44 (72.1)	0.737	164 (74.5)	23 (82.1)	0.518
Preexisting illness, n (%)						
Previous PCI history	41 (5.7)	1 (1.6)	0.288	5 (2.3)	1 (3.6)	1.000
Hypertension	264 (36.9)	15 (24.6)	0.074	59 (26.8)	7 (25.0)	1.000
Diabetes	164 (22.9)	12 (19.7)	0.671	43 (19.5)	2 (7.1)	0.179
Renal impairment	13 (1.8)	0 (0.0)	0.588	2 (0.9)	0 (0.0)	1.000
Cerebrovascular accident	12 (1.7)	0 (0.0)	0.632	2 (0.9)	0 (0.0)	1.000
Trauma mechanism			1.000			1.000
Blunt	712 (99.6)	61 (100.0)		219 (99.5)	28 (100.0)	
Penetrating	3 (0.4)	0 (0.0)		1 (0.5)	0 (0.0)	
Revised trauma score	5.97 (5.64–7.84)	4.09 (2.83–5.97)	< 0.001	5.97 (5.08–7.84)	4.09 (2.63–5.03)	< 0.001
Injury severity score	17 (16–25)	25 (20–25)	< 0.001	26 (22–34)	29 (23–38)	0.066
Glasgow Coma Scale score	14 (8–15)	4 (3–6)	< 0.001	15 (8–15)	3 (3–7)	< 0.001
Systolic BP, mmHg	130 (110–150)	140 (105–160)	0.232	110 (100–130)	85 (70–110)	< 0.001
Respiratory rate, /min	20 (20–20)	20 (20–22)	0.229	20 (20–22)	22 (20–24)	0.002
Pulse rate, /min	82 (72–92)	90 (72–105)	0.022	90 (80–104)	97 (80–110)	0.244
Body temperature, °C	36.4 (36.2–36.8)	36.2 (36.0–36.5)	< 0.001	36.4 (36.0–36.8)	36.2 (36.0–36.3)	0.003
Massive transfusion, n (%)	33 (4.6)	7 (11.5)	0.043	31 (14.1)	14 (50.0)	< 0.001
Emergency operation, n (%)	206 (28.8)	10 (16.4)	0.054	37 (16.8)	6 (21.4)	0.732

*TBI* traumatic brain injury, *BP* blood pressure

**Table 4:** Comparison of electrocardiogram variables according to in-hospital mortality within 48 h between the isolated TBI and combined TBI groups

Variables	Isolated TBI (*N* = 776)			Combined TBI (*N* = 248)		
	Survivors (*n* = 715)	Non-survivors (*n* = 61)	*P*	Survivors (*n* = 220)	Non-survivors (*n* = 28)	*P*
PR, ms	166 (152–188)	162 (138–176)	0.010	152 (136–172)	138 (132–172)	0.080
PR prolongation, n (%)	114 (15.9)	8 (13.1)	0.690	20 (9.1)	1 (3.6)	0.530
QRS, ms	92 (86–102)	92 (82–102)	0.342	89 (82–100)	91 (83–117)	0.272
QRS widening, n (%)	50 (7.0)	7 (11.5)	0.302	14 (6.4)	6 (21.4)	0.017
QTc, ms	458 (438–480)	476 (454–513)	< 0.001	462 (444–482)	484 (466–511)	0.002
QTc prolongation, n (%)	180 (25.2)	28 (45.9)	< 0.001	65 (29.5)	15 (53.6)	0.019
Presence of STE, n (%)	55 (7.7)	9 (14.8)	0.093	16 (7.3)	4 (14.3)	0.360
Presence of STD, n (%)	85 (11.9)	30 (49.2)	< 0.001	23 (10.5)	20 (71.4)	< 0.001

*TBI* traumatic brain injury, *STE* ST-segment elevation, *STD* ST-segment depression

In the combined TBI group, survivors had higher RTS, GCS score, SBP, and BT values and lower RR value than non-survivors (Table 3). The QTc interval and incidence rates of QRS widening, QTP, and STD were significantly different between survivors and nonsurvivors (Table 4).

### Multivariate analysis for predicting 48-h mortality in patients with TBI

After adjusting for confounding factors, RTS (OR, 0.561; 95% CI, 0.483–0.650), BT (OR, 0.627; 95% CI, 0.413–0.953), emergency operation (OR, 0.363; 95% CI, 0.191–0.688), QTP (OR, 2.017; 95% CI, 1.203–3.382), and STD (OR, 8.428; 95% CI, 5.019–14.152) were independently associated with 48-h mortality in patients with TBI (Table 5).

**Table 5:** Multivariate logistic regression analysis for predicting in-hospital mortality within 48-h in patients with TBI

Variables	Adjusted OR (95% CI)	*P*
Age, years	1.013 (0.996–1.031)	0.127
Hypertension	0.664 (0.370–1.191)	0.170
Revised trauma score	0.561 (0.483–0.650)	< 0.001
Injury severity score	1.010 (0.972–1.049)	0.620
Pulse rate, /min	1.007 (0.994–1.020)	0.281
Body temperature, °C	0.627 (0.413–0.953)	0.029
Massive transfusion	1.797 (0.875–3.691)	0.111
Emergency operation	0.363 (0.191–0.688)	0.002
ECG variables		
PR prolongation	0.625 (0.277–1.412)	0.259
QRS complex widening	1.584 (0.707–3.547)	0.264
QTc prolongation	2.017 (1.203–3.382)	0.008
Presence of STE	1.079 (0.454–2.563)	0.863
Presence of STD	8.428 (5.019–14.152)	< 0.001

*TBI* traumatic brain injury, *OR* odds ratio, *CI* confidence interval, *ECG* electrocardiogram, *STE* ST-segment elevation, *STD* ST-segment depression

In the isolated TBI group, QTP (OR, 2.098; 95% CI, 1.111–3.962) and STD (OR, 5.903; 95% CI, 3.146–11.076) were independently associated with 48-h mortality. In the combined TBI group, QTP (OR, 2.837; 95% CI, 1.011–7.958) and STD (OR, 15.430; 95% CI, 5.528–43.067) were associated with 48-h mortality (Table 6).

**Table 6:** Multivariate logistic regression analysis for predicting in-hospital mortality within 48 h between the isolated TBI and combined TBI groups

	Isolated TBI group		Combined TBI group	
	Adjusted OR (95% CI)	*p*	Adjusted OR (95% CI)	*p*
Age, years	1.010 (0.989–1.031)	0.349		
Hypertension	0.509 (0.251–1.030)	0.060		
Diabetes			0.394 (0.057–2.720)	0.345
Revised trauma score	0.563 (0.473–0.669)	< 0.001	0.586 (0.422–0.814)	< 0.001
Injury severity score	1.076 (1.005–1.152)	0.035	0.963 (0.899–1.031)	0.279
Pulse rate, /min	1.012 (0.996–1.027)	0.132		
Body temperature, °C	0.710 (0.435–1.158)	0.170	0.555 (0.213–1.447)	0.229
Massive transfusion, n (%)	1.364 (0.434–4.285)	0.596	2.484 (0.850–7.256)	0.096
Emergency operation	0.200 (0.089–0.448)	< 0.001		
ECG variables				
PR prolongation	0.648 (0.262–1.604)	0.348	1.618 (0.159–16.487)	0.684
QRS complex widening	1.553 (0.564–4.276)	0.394	1.666 (0.346–8.008)	0.524
QTc prolongation	2.098 (1.111–3.962)	0.022	2.837 (1.011–7.958)	0.048
Presence of STE	1.642 (0.605–4.461)	0.330	0.278 (0.052–1.473)	0.132
Presence of STD	5.903 (3.146–11.076)	< 0.001	15.430 (5.528–43.067)	< 0.001

*TBI* traumatic brain injury, *OR* odds ratio, *CI* confidence interval, *ECG* electrocardiogram, *STE* ST-segment elevation, *STD* ST-segment depression

### Prognostic performance of ECG variables for 48-h mortality in patients with TBI

**Fig. 2:**
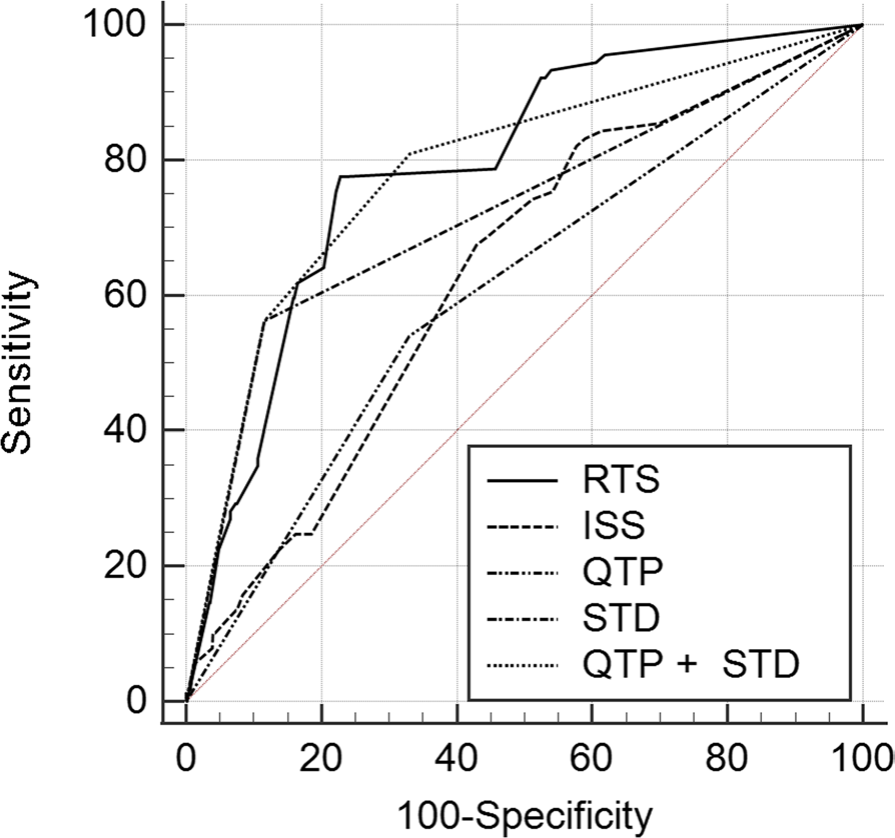
Receiver operating characteristic curve analyses of the RTS, ISS, QTP, STD, and the combination of QTP and STD for predicting 48-h mortality in patients with TBI. The AUCs of RTS, ISS, QTP, STD, and the combination of QTP and STD were 0.790 (95% CI, 0.764–0.815), 0.632 (95% CI, 0.602–0.662), 0.605 (95% CI, 0.574–0.635), 0.723 (95% CI, 0.695–0.750), and 0.786 (95% CI, 0.759–0.811), respectively. The AUC of the combination of QTP and STD was significantly different from that of ISS, STD, and QTP, but not RTS. RTS, revised trauma score; ISS, injury severity score; QTP, prolongation of corrected QT interval; STD, ST-segment depression; AUC, area under the curve; CI, confidence intervals

On the basis of multivariate analysis, we set a limit to 48-h mortality in patients with TBI. The presence of STD (4 points) and/or QTP (1 point) was considered to predict 48-h mortality in patients with TBI. When QTP and STD were combined, the sum of scores ranged from 0 to 5, in which a higher score indicated a higher likelihood of 48-h mortality. The AUCs of RTS, ISS, QTP, STD, and the combination of QTP and STD were 0.790 (95% CI, 0.764–0.815), 0.632 (95% CI, 0.602–0.662), 0.605 (95% CI, 0.574–0.635), 0.723 (95% CI, 0.695–0.750), and 0.786 (95% CI, 0.759–0.811), respectively (Fig. 2). The AUC of the combination of QTP and STD was significantly different from that of ISS, STD, and QTP, but not RTS.

## Discussion

Among the ECG variables, QTP and STD were independently associated with 48-h mortality in patients with TBI. The combination of QTP and STD had a similar performance to RTS in predicting 48-h mortality in patients with TBI. In both the isolated and combined TBI groups, QTP and STD were associated with 48-h mortality.

Krishnamoorthy et al. suggested that QTP (QTc > 440 ms) is related to cardiac dysfunction, including ejection fraction < 50% or regional wall motion abnormality on ECG, in patients with isolated TBI [22]. In patients with traumatic subarachnoid hemorrhage (SAH), the QTc interval was related to the severity of SAH based on computed tomography [18]. In another study on TBI, the QTc intervals of nonsurvivors were significantly more prolonged than those of survivors at hospital admission, consistent with the present study findings [23]. With respect to abnormal ECG findings in patients with TBI, the mechanism by which the brain affects the heart may be paroxysmal sympathetic hyperactivity [6]. In patients with SAH, the catecholamine level in cerebrospinal fluid was correlated with the severity of SAH [9].

Enhanced sympathetic activity can also induce ST-segment changes, including STE and STD, in severe neurological impairments such as TBI, SAH, or refractory seizures [24]. In a study on patients with SAH, 15% of patients with preoperative SAH developed STD [25]. ST-segment abnormalities were the most commonly reported ventricular repolarization disorders in TBI [26]. Even in the present study, STD occurred in 15.4% of the total patients with TBI, and the proportion of patients with STD with poor outcome was higher than that of patients with good outcome. Several studies have investigated the association between STD and the severity of head injury. In pediatric patients with TBI, STD led to hemodynamic instability or cardiac arrest, which improved only after surgery or other procedures [27]. In aneurysmal SAH, STD appears to have a significant relationship to the neurological outcome [28]. Manninen et al. [29] reported that the incidence of ECG abnormalities was statistically higher in patients with increased amounts of intracranial blood or intracerebral clots observed on computed tomography. STD in TBI is considered to cause myocardial ischemia due to severe sympathetic stimulation and elevated intracranial pressure.

Some studies have assessed the occurrence of STE in TBI [30–32]. However, none of the studies evaluated the frequency of STE or its association with outcomes such as mortality in TBI. Similar to STD, STE can also cause coronary vasospasm along with myocardial dysfunction with elevated intracranial pressure and consequent sympathetic activation. These sequential effects may eventually influence the outcome of TBI. In the present study, the proportion of patients with STE was higher among nonsurvivors than among survivors; however, there was no significant association with 48-h mortality in multivariate analysis. In the case of SAH, STE appeared to be related to delayed cerebral ischemia, although this relationship is still controversial [33]. Prospective multicenter studies on STE are needed to elucidate this issue.

In the present study, RTS was associated with 48-h mortality in TBI. We considered that the GCS score, a component of RTS, plays an important role in predicting 48-h mortality. Several studies have demonstrated that the GCS score is related to mortality in patients with TBI [34, 35]. In a study by Han et al., a GCS score of ≤5 was associated with mortality in TBI, and the GCS score of nonsurvivors in the present study was 4 (3–9) [35]. However, there are several barriers to determining the GCS score. The verbal GCS score may show variability in intubated patients, and the GCS score may be influenced by other factors that affect the level of consciousness, such as alcohol or sedative use. In addition, the reliability of the GCS score evaluated immediately after resuscitation is controversial. In contrast, in ECG measurement, there is no difference in score between evaluators, the effect of alcohol or sedatives is less than that on the GCS score, and the ECG results are hardly affected by different procedures.

This study had some limitations. First, because this was a retrospective study conducted in a single center, our results cannot be immediately generalized to the entire population. Further multicenter studies with larger sample sizes and a prospective design are needed to substantiate our findings. Second, we did not obtain data for all ECG variables in patients with TBI. However, as there was no difference in mortality between patients for whom ECG was available and those who did not (8.7% vs. 14.3%; *p* = 0.087), we can conclude that ECG abnormalities frequently occur in patients with severe TBI and that ECG abnormalities are associated with 48-h mortality. Third, we included ECG data alone at ED arrival. Thus, we could not investigate the serial changes in ECG findings or timing of ECG measurement that best reflects the prognosis of TBI. Fourth, we are unsure whether all potential factors, which can cause ECG changes, such as hypotension or hypothermia, were excluded. Although the confounding factors including systolic BP and hypothermia determined based on RTS and BT were adjusted, the factors that were overlooked may have influenced the ECG changes. For example, plasma hyperosmolarity was associated with QTP and atrial fibrillation [36]. In addition, disorders in electrolyte ions, including calcium and sodium, can influence the ECG changes including QTP or ST segment change [37, 38]. Fifth, we did not analyze the effect of medication history in patients with TBI. Data about the medication history were inaccurate and insufficient; hence, it could not be included in the analysis of our study. Finally, external or internal validation was not performed in this study. Thus, the ECG results including QTP and STD were not considered as predictors of the outcome of patients with TBI. In previous studies that focused on the phenomenon of ECG change after TBI, our study demonstrated the association between the acute phase prognosis of TBI and ECG in terms of 48-h mortality. It may be difficult to directly apply it to clinical practice. However, we believe that at least QTP and STD could reflect the condition of patients with TBI, which could serve as basis for developing the treatment guidelines.

## Conclusion

In this study, QTP and STD were independently associated with 48-h mortality in patients with TBI. The combination of QTP and STD had a similar performance to RTS in predicting 48-h mortality. Based on the ECG findings, QTP and STD were associated with 48-h mortality in patients with TBI.

## Data Availability

The data used and analyzed during the current study are available from the corresponding author on reasonable request.

## Abbreviations

TBI: Traumatic brain injury
ISS: Injury severity score
RTS: Revised trauma score
ECG: Electrocardiogram
ED: Emergency department
AIS: Abbreviated injury scale
RR: Respiratory rate
PR: Pulse rate
BT: Body temperature
GCS: Glasgow Coma Scale
SBP: Systolic blood pressure
MT: Massive transfusion
QTc: Corrected QT interval
QTP: QTc prolongation
STE: ST-segment elevation
STD: ST-segment depression
OR: Odds ratio
CI: Confdence interval
AUC: Area under the curve
